# Supplementary material for: Common knowledge processing patterns in networks of different systems
Source: PLoS One. 2023 Oct 5;18(10):e0290326. doi: 10.1371/journal.pone.0290326 (PMC10553345; doi:10.1371/journal.pone.0290326)
Supplement: Attachment — Submitted filename: Response to Reviewers.pdf [file pone.0290326.s001.pdf]

Editor’s comments

2. In your Data Availability statement, you have not specified where the minimal data set underlying the results described in your manuscript can be found. PLOS defines a study's minimal data set as the underlying data used to reach the conclusions drawn in the manuscript and any additional data required to replicate the reported study findings in their entirety. All PLOS journals require that the minimal data set be made fully available. For more information about our data policy, please see http://journals.plos.org/plosone/s/data-availability.

Response:

The availability of existing dataset section, which appears at the end of the paper, lists all the datasets used for the experiment. The conclusion of this manuscript was drawn from comparative analysis between the datasets. The data availability statement has been updated as follows to better reflect the dataset availability and the reason for multiple data points. The minimal data set is the summarization of such dataset, which is uploaded to a public repository.

- “Combinations of existing and generated datasets in multidisciplinary fields were used in the experiment. The authors decided to keep the datasets separate instead of having them in a single repository based on the various natures of the datasets being used, and therefore the datasets can be found in multiple locations including their original sources. All the datasets used in the experiment are publicly available.”
- “Three existing multilabel classification datasets were used for reviewing the Patterns of Decision Making in Artificial Neural Networks, where a series of images are classified into a set of labels. They are all publicly available.
    - MNIST (http://yann.lecun.com/exdb/mnist/): Handwritten digits
    - Fasion_MNIST (https://github.com/zalandoresearch/fashion-mnist): Fashion items
    - KMNIST (http://codh.rois.ac.jp/kmnist/index.html.en): Hiragana (Japanese characters)”
- “Analysis of the Information Processing Mechanism on the Academic Topic Networks are done on eight journal-specific datasets curated from the Microsoft Academic Graph bibliography database. The curated dataset is made public in the following location.
    - Domain-specific Topic Co-occurrence Networks from Microsoft Academic Graph

  (https://zenodo.org/record/6547761).”
- “Similar Patterns in Randomly Generated Graphs were reviewed from fixed graphs generated for the experiment. The generated graphs were recorded online for public access.
    - Four random graph generation algorithms with example graph binary files (https://zenodo.org/record/6547767).”

- “The Knowledge Processing Patterns in Animal Neuron-Synapse Structures were analyzed based on two existing neural map connectomes, which were made public by respective research publications.
    - Roundworm connectome (http://www.wormatlas.org/handbook/nshandbook.htm/nswiring.htm): Outdated, from Paper "Wiring optimization can relate neuronal structure and function".
    - Fruit fly hemisphere connectome (https://neuprint.janelia.org/): From Paper "A connectome of the adult drosophila central brain".”
- Lastly, the minimal data set is uploaded to an online data repository for public access.
    - Common Knowledge Processing Patterns in Networks of Different Systems Minimal Data Set (https://zenodo.org/record/8122402).

I would like to add that more rigid statistical validation is required in order to prove robustness of your approach. Please choose carefully adequate parametric or non-parametric statistical test.

Response:

The “Results” section has been augmented with statistical comparisons to show a more objective form of analysis and comparison. The following statements are added to the various paragraphs in the section.

In the second paragraph, for graph learning characteristics:

- “; the correlation coefficient of 0.4322 between the accuracy the epoch count of up to 10 show medium correlation during the initial stage of network maturation.”
- “The smaller changes are also reflected on its jagged patterns, which shows nearly no correlation (-0.0566) and statistically significant mean value differences (p=0) with the accuracy. Similarly, all other measures showed low to no correlation to the accuracy with the clustering showing the highest value (0.2533). This indicates that the maturity of the knowledge processing network is not governed by a single graph structure measure.”
- “along with the degree (b) over epochs with near identical patterns (correlation value of 0.9963, with statistically significant mean normalized differences with p=0). This is a natural behavior of growing networks as the change in the average degrees directly effects the average number of triangles each node can be a member of.”

In the third paragraph, for topic behaviors in an “invisible brain”:
- “with extremely high correlation (0.9755).”
- “While the degree and the triangles show the same high correlation (0.9267) as they did in the previous example, they show an inverse pattern over time with

increasing values as opposed to in Figure 1. This represents a different knowledge processing pattern; while the classification ANNs matured by disseminating their functions over network structures, journal publications instead matured by centralizing on key topics. Such differences can be seen between the relationship between the four evaluation measures and their sizes as well; while ANOVA shows statistically significant differences ($p = 0$) between all six variables, their evolution pattern show different similarities with diminishing PageRank and clustering values over the years."

No statistical analysis is added to the fourth paragraph as it discusses the characteristics of randomly generated graphs. In the fifth paragraph for animal connectome datasets, the following texts have been added to explain the analysis in more detail.

- "artificially generated for each animal in Table 1 using ten uniformly distributed edge completion ratio ranging from p(fruitfly) = 0.012164 to p(worm) = 0.061134. With their estimated number of neurons as the number of nodes, edges between the neurons are generated using the ratio values ($p_1, p_2, \ldots, p_{10}$) to reflect the varying degree of connectome complexity."
- "As the number and distribution of edges are governed by a graph generation algorithm, all three evaluation measures used in the analysis showed statistically insignificant differences with ANOVA p-value of 1 across the eight animals, indicating that the main factor is the number of neurons."

Reviewer #1:

1. Avoid using we/our throughout the paper.

Response:

The entire paper was rephrased to avoid using we/our.

2. Third sentence in the Introduction: Can there be... is not clear, please rephrase it.

Response:

The sentence was rephrased:

> "Can there be an underlying structure that repeats across systems, when these systems process information for different purposes and seem to have different infrastructures?"

3. Fourth paragraph in the Introduction, starting with What if we compare... if those are questions, please add question marks (?)

Response:

Yes, these are questions. The punctuation was changed accordingly:

> “What if artificial neural network knowledge processing patterns are compared with knowledge processing patterns of a variety of animals that show different complexity of knowledge processing patterns, or in other words different brain complexity? Would there be any common characteristics?”

4. State the research question, and list the main contributions clearly in the Introduction.

Response:

The research question and main contributions were added to the introduction:

“Biology has DNA as an underlying basic element characteristic, Chemistry has the Periodic Table, and Physics has a set of equations. This work analyzes whether knowledge has an underlying structure, “DNA for Knowledge”.

The main contributions are:

- A new knowledge modeling approach is presented based on evolving graphical characteristics.
- The knowledge modeling approach is analyzed on different network processing systems: artificial neural networks, academic topic networks, graph generation algorithms, and animal neuron-synapse structures.
- From the different graphical evolving characteristics some similarities appear across the different network processing systems.”

5. Provide paper structure at the end of introduction.

Response:

The paper structure was added to the end of the Introduction section:

> “The next section provides background information on related work on knowledge processing in neuronless, non-biological systems and is followed by the methods section describing the knowledge processing comparison analysis and the experiments on different network processing systems. Then the results section identifies the characteristics that appear in the different processing systems. The discussion section presents the overall commonality of the knowledge model across the processing systems. Finally, the conclusion summarizes the results and presents future work.”

6. The literature survey should include the swarm intelligence/machine learning section, as it is considered to be the state of the art. You can include the following:
https://www.mdpi.com/2075-1680/12/3/266
https://onlinelibrary.wiley.com/doi/abs/10.1002/cpe.6629
https://www.sciencedirect.com/science/article/pii/S2210670720308842
https://dl.acm.org/doi/abs/10.1145/3546194
https://link.springer.com/chapter/10.1007/978-981-16-5348-3_54

Response:

A new subsection was added which includes all the references suggested:

> **“Machine Learning and Nature-Inspired Algorithms**
>
> Machine learning has been extensively used in areas such as content-based medical image retrieval [20], topic classification of online news articles [21], and prediction of solar energy generation [22]. Multiple approaches have been used to simplify the processing of the neural network. A swarm intelligence-based classification algorithm was proposed for reducing dimensionality (feature selection) in datasets [23]. Other approaches use heterogeneous graph embedding to learn the low-dimensional representations of nodes, such as link prediction, node classification, and community detection [24]. Furthermore, community detection was performed by mapping nodes into communities based on a random walk in the network [25].
>
> Nature-inspired machine learning algorithms appear in characterization of abnormalities in breast cancer images involving training a Convolutional Neural Network using genetic algorithm (GA), whale optimization algorithm (WOA), and multiverse optimizer (MVO), satin bower optimization (SBO), and life choice-based optimization (LCBO) algorithms to optimize weights and bias of the model [26]. In addition, COVID-19 cases prediction was performed using a hybrid machine learning and beetle antennae search approach [27].”

7. The experiments should be elaborated in more details.

Response:

More experiment descriptions are added to the manuscript as per the feedback to Reviewer #2's comment on clearer explanation of methods.

The two additional measures for ANNs are better explained by modifying the last three paragraphs in the section "Patterns of Decision Making in Artificial Neural Networks" as follows.

> "The shared patterns shown during the decision-making process on different ANNs are analyzed. For each data set the learning neural network characteristics was analyzed using the four graph evaluation measures; degree, PageRank, clustering, and triangles. The measures are averaged across each graph to generate a single value for any given graph. With multilabel classification datasets, the classification accuracy is recorded to show whether the found patterns are correlated to the correct decision-making process. Cohesion is also added as a relative quality measure for a set of subgraphs within the whole graph *G* ($G' = \{V', E'\}$ where $E' = \{e_{i,j} | v_i, v_j \in V'\}$). It is defined as the relative quality of the subgraphs to the whole graph *G* or the ratio of edges internal to the subgraph $|E'|/\{e_{i,j} | v_i \in V \cup v_j \in V, output_t(v_i|v_j) > 0.1\}$. The threshold variable $output_t(v_i|v_j) > 0.1$ is included in the formula to differentiate the otherwise complete graph; only nodes with output greater than the *graph_threshold = 0.1* are considered as the external neurons. Finally, the degree of knowledge processing maturity is captured by running with a different number of epochs during the ANN training to represent varying degree of training and network maturity. A total of 100 epochs are used for each dataset, and knowledge patterns at epochs up to 10, and every 10th up to 100 are recorded."

8. The purpose of the experiments should be highlighted.

Response:

The following has been added at the end of the "Invisible Brain" section to signify the purpose of the experiment.

> "The work studied four knowledge processing patterns by accessing the structural patterns of natural and artificial networks responsible for such activities. This explorative study aims to test whether neuron networks responsible for knowledge processing in living organisms have any commonalities with manufactured networks with or without knowledge processing capabilities."

9. The discussion is very limited, it should be more elaborate.

Response:

The discussion was reorganized and expanded:

- “The overall result analysis shows an increase in the average degree, which measures the number of edges compared to the number of nodes, and an increase in the average number of triangles, measuring the ratio of smallest cliques in the network. This shows that as more knowledge is represented by the network the network becomes more tightly knitted.

  At the same time there is a decrease in the PageRank, which measures the average importance of the connected nodes in the network, and a decrease in the average clustering, which measures node clusterability based on the number of triangles each node is a member of. These results show that specific nodes become less unique or important as the knowledge in the network increases and the nodes representing specific knowledge are more widely shared or less clustered together as the network increases. “

10. The conclusions should be clear and backed up with experimental results

Response:

In addition to the clarification of the experimental results described above, a Conclusion and Further Research section was added:

“Conclusion and Further Research

Basic patterns of knowledge processing are found across systems which appear to be essentially different and each one serves a different purpose. Furthermore, some of these systems are naturally created biologically based systems, while others are artificial systems created by humans and work on electronic circuits. The similarity in patterns of knowledge processing systems raises the question of how unique are humans in processing knowledge.

Further research can improve the similarity in values of properties such as clustering by building more tailored algorithms for representing the graph growth as the knowledge represented increases. Other artificial neural networks might fit the patterns found in topic networks, random graph generation methods, and mapping animals with increasing number of neurons. Analyzing convolution neural networks, deep neural networks, and graph neural networks for these characteristics might yield interesting results. In addition, as additional research

works identifying the sizes of different animal neural networks increase, verifying the different animal neural network sizes, the properties projecting the graph characteristics can be adjusted."

Reviewer #2:

• More in-depth analysis of related works. Add a separate Related Works section to discuss as currently it is missing. Here are some suggestions to start with: Topic classification of online news articles using optimized machine learning models. Computers, 12(1); Relation-aware weighted embedding for heterogeneous graphs. Information Technology and Control, 52(1), 199-214. Large scale community detection using a small world model. Applied Sciences, 7(11).

Response:

A Related Work section was added along with a subsection containing all the references suggested:

"Related Work

Machine Learning and Nature-Inspired Algorithms

Machine learning has been extensively used in areas such as content-based medical image retrieval [20], topic classification of online news articles [21], and prediction of solar energy generation [22]. Multiple approaches have been used to simplify the processing of the neural network. A swarm intelligence-based classification algorithm was proposed for reducing dimensionality (feature selection) in datasets [23]. Other approaches use heterogeneous graph embedding to learn the low-dimensional representations of nodes, such as link prediction, node classification, and community detection [24]. Furthermore, community detection was performed by mapping nodes into communities based on a random walk in the network [25].

Nature-inspired machine learning algorithms appear in characterization of abnormalities in breast cancer images involving training a Convolutional Neural Network using genetic algorithm (GA), whale optimization algorithm (WOA), and multiverse optimizer (MVO), satin bower optimization (SBO), and life choice-based optimization (LCBO) algorithms to optimize weights and bias of the model [26]. In addition, COVID-19 cases prediction was performed using a hybrid machine learning and beetle antennae search approach [27]."

• Clearer Explanation of Methods: While your methodology is intriguing, a more detailed explanation of the procedures used, such as the graph analysis techniques and the criteria for comparing different systems, would enhance the understanding and replicability of your research.

Response:

The graph analysis was carried out with the same evaluation measures over all datasets since artificial networks are all preprocessed to share the same graph format as the biological networks. The comparative analysis is done manually by evaluating the structures of individual graphs and interpreting commonalities and differences in their patterns. The following is added at the start of the “Results” section to signify the comparative nature of the experiment.

- “The generated graphs are analyzed to detect unique and shared characteristics between biological and artificial knowledge processing networks, with the random networks as a baseline with the simplistic nature and the connectomes as the biological structure for the knowledge processing functions. Each graph is first analyzed independently with the evaluation measures, and the commonalities and differences in their measures are then reviewed. Artificial networks with similar evaluation measures to biological networks are interpreted as networks sharing knowledge processing mechanisms to the neural and brain networks.”

The considerations needed for comparing different systems are embedded into the data preprocessing phase, which is shown by adding the following paragraph at the end of the “Methods - Knowledge Processing Comparison Analysis” section.

- “The evaluation measures provide domain-independent quality characteristic information for any graphs they are applied on. All the datasets used in the experiment share an identical graph format, therefore the measures can be used to objectively compare the characteristics of networks with vastly different origins. To achieve this, preprocessing has been performed on some of the artificial networks such as the Artificial Neural Networks which are usually fully connected to begin with. After the dataset preprocessing and graph generations, comparison between structures of different domains is reduced to a problem of comparison between different directed graphs.”

• Discussion on Degree Consistency: Your observation that artificial neural networks align with most trends in graph generation algorithms regarding PageRank, clustering, and triangles but not degree is interesting. A more in-depth discussion about the implications of this inconsistency would be useful.

Response:

A stark difference seen in the degree is because of the nature of graph generation algorithms being used; three out of the four algorithms show near-static degree values since the algorithms are designed to achieve such effects. The only outlier (G(n,p)) generates the graph with a pre-set edge probability variable, which linearly increases the average degrees as the possible edge population grows exponentially with each expansion. The descriptions below are added to explain the reason in the manuscript.

- “This is because only Gn,p has a edge generation variable dependent on the population sizes. The BA model dictates the degree with the existing node connection variable e, which results in edge overlaps in lower iterations. Gn,m and Gn,d each dictate their edge generations with a fixed edge size e and regular graph with neighbor count d, resulting in a static degree during graph expansion. Only Gn,p results in a linear growth in the degree as the population of possible edges increase exponentially. The number of edges therefore increase exponentially with static edge probability p resulting in linear increase in node degrees.”

The discussion was reorganized and expanded:

> “The overall result analysis shows an increase in the average degree, which measures the number of edges compared to the number of nodes, and the average number of triangles, measuring the ratio of smallest cliques in the network. This shows that as more knowledge is represented by the network the more tightly knitted the network becomes.
>
> While at the same time there is a decrease in the PageRank, which measures the average importance of the connected nodes in the network, and the average clustering, which measures node clusterability based on the number of triangles each node is a member of. These results show that specific nodes become less unique or important as the knowledge in the network increases and the nodes representing specific knowledge are more widely shared or less clustered together as the network increases. “

• Future Directions: In the conclusion, you mention potential areas for further research. Fleshing out these ideas a little more would provide an excellent direction for future work in this field.

Response:

In addition to the clarification of the experimental results described above, a Conclusion and Further Research section was added:

> **“Conclusion and Further Research**
>
> Basic patterns of knowledge processing are found across systems which appear to be essentially different and each one serves a different purpose. Furthermore, some of these systems are naturally created biologically based systems, while others are artificial systems created by humans and work on electronic circuits. The similarity in patterns of knowledge processing systems raises the question of how unique are humans in processing knowledge.
>
> Further research can improve the similarity in values of properties such as clustering by building more tailored algorithms for representing the graph growth as the knowledge represented increases. Other artificial neural networks might fit the patterns found in topic networks, random graph generation methods, and mapping animals with increasing number of neurons. Analyzing convolution neural networks, deep neural networks, and graph neural networks for these characteristics might yield interesting results. In addition, as additional research works find the sizes of different animal neural networks, verifying the different animal neural network sizes, the properties projecting the graph characteristics can be adjusted.”
